# Visualization and identification of components in a gigantic spherical dolomite concretion by Raman imaging in combination with MCR or CLS methods

**DOI:** 10.1038/s41598-024-51147-y

**Published:** 2024-01-07

**Authors:** Ryosuke Kitanaka, Motohiro Tsuboi, Tomoko Numata, Yusuke Muramiya, Hidekazu Yoshida, Yukihiro Ozaki

**Affiliations:** 1 Department of Applied Chemistry for Environment, School of Biological and Environmental Sciences, Kwansei Gakuin University, 1 Gakuen Uegahara, Sanda, Hyogo 669-1330 Japan; 2 HORIBA, Techno Service Co., Ltd., Chiyoda, Tokyo 101-0063 Japan; 3 Fukada Geological Institute, 2-13-12 Honkomagome, Bunkyo-ku, Tokyo, 113-0021 Japan; 4 Material Research Section, Nagoya University, University Museum, Chikusa, Nagoya 464-8601 Japan; 5 Department of Biomedical Sciences, School of Biological and Environmental Sciences, Kwansei Gakuin University, 1 Gakuen Uegahara, Sanda, Hyogo 669-1330 Japan

**Keywords:** Analytical chemistry, Geochemistry, Geology, Palaeontology, Petrology

## Abstract

The combination of Raman imaging and multivariate curve resolution (MCR) or classical least squares (CLS) has allowed us to explore the distribution and identification of components in a gigantic spherical dolomite concretion. It has been found by the MCR and CLS analyses of imaging data that the concretion contains dolomite, kerogen, anatase, quartz, plagioclase, and carbon materials with considerably large distribution of dolomite. The existence of these components has also been confirmed by the point-by-point analysis of imaging data. The distributions of these components were clearly observed by Raman images. Of note is that the amount of carbon materials is considerably large, and they are buried among the matrix sedimentary grains in the concretion, suggesting that there exist soft tissues with biological origin. Moreover, one of the loading spectra of CLS shows intense bands in the region of 3000–2800 cm^−1^, and bands at ca. 1658, ca. 1585, 1455, 1323, and 1261 cm^−1^. These bands indicate the existence of decomposed organic materials in the concretion. Raman imaging of concretions provides direct evidence that concretions are of biological organic origin.

## Introduction

The purpose of the present study is to investigate the identification, visualization and distributions of components in a concretion by use of Raman imaging in combination with MCR or CLS analysis. To our best knowledge the combination of Raman imaging with MCR or CLS may be the first time for the fields of earth science (mineralogy). From the results of Raman imaging we have looked for clear evidences for the existence not only of dolomite, kerogen and usual mineral components but also of soft tissues with biological origins. Based on the findings of the existence of these materials including biological materials we explored the mechanism of the formation of concretion. The present study also has aimed at demonstrating potential of new analysis methods of Raman imaging with chemometrics.

One can find spherical concretions mainly in marine sedimentary rocks worldwide^1–9^.

The concretions are composed mainly of carbonates and matrix sedimentary grains, and they sometimes contain well-preserved fossils such as ammonites or others in their interior^1–3^. The size of the concretions varies from a few millimeters to more than 1 meter^4,5^ and fossil source organisms are considered to be responsible for the formation of the concretions. Although concretion formation models have not been studied in detail, Yoshida et al.^5,10,11^ recently conducted a systematic study of concretions and proposed a new model for their formation mechanism and duration.

According to their proposal^5,10,11^, the results showed that concretions can be formed in months to years, although it was previously considered that concretions formed on the order of tens of thousands to millions of years. Muramiya et al.^4^ studied the formation mechanism and rate of formation of giant concretions and found that they were formed by processes similar to those of smaller concretions and at a faster rate, within 10 years. Yoshida et al.^5^ also showed that the organic matter in the concretion was of biogenic origin by the analysis of carbon isotope (δ^13^C), and that the concretion was formed by bicarbonate ions supplied by the biological remains and calcium ions supplied by the seawater.

Raman spectroscopy has recently been used for the studies of fossils^12,13^, however, its application to concretions might be very rare. Raman spectroscopy allows a nondestructive analysis for various materials including minerals^14,15^ and biomedical samples^16,17^. It does not require troublesome sample preparation in most cases, and spectra can be obtained in situ. Moreover, recent progress of micro Raman spectroscopy and imaging has enabled Raman measurements and imaging in a microscopic range (500 nm to 1 μm), making Raman spectroscopy more useful^16–19^.

In our previous study, concretions with and without fossils were investigated by micro-Raman spectroscopy in a nondestructive manner^12^. To investigate the origin of apatite in the concretions the band position and full width at half maximum height (FWHM) of ν_1_-PO_4_^3−^ band of apatite were analyzed. The micro-Raman analysis showed that the apatites in the concretions could be categorized into two groups:^12^ Group W with a wide FWHM and Group N with a narrow FWHM. It has been suggested that the apatite in Group W originated from the soft body tissues of organisms. According to this Raman spectroscopic study, the apatite was initially from Group W during concretion formation but was changed to Group N due to the substitution of F during the diagenesis process^12^.

Gigantic concretion occurs in the tuffaceous sandstone of the Miocene Morozaki Group in Chita Peninsula, southwestern Japan, which has been studied in detail by Muramiya et al.^4^. In this study, we have used Raman imaging on this concretion. It is not always a novel technique but its recent development is really remarkable^16–19^. Raman imaging has been used extensively in a variety of research fields such as materials science, biomedicine, nanoscience and technology, pharmaceutical engineering, and food technology^16–19^. Minerals have recently become important targets of Raman imaging^20,21^. One can use this technique for visualizing the distributions of components but also those for physical properties such as crystallinity. Recently, several new decomposition methods of Raman images have been proposed; for example, MCR and CLS. These have been used mainly for the analysis of Raman imaging of biomedical samples^16,17^. In this paper we demonstrate the potential of these spectra decomposition methods in the study of concretion. Raman imaging may allow us to explore mechanism of the formation of concretion in situ.

## Materials and methods

### Materials

The sample was taken from a gigantic spherical dolomite concretion in the Miocene Morozaki Group, Chita Peninsula, southwestern Japan (Fig. SI 1). Figure SI 2 shows the gigantic spherical concretion investigated. The diameter of the concretion was approximately 170 cm. We obtained the core sample from the center of the concretion. A red arrow in Fig. SI 2 indicates a drilled point. We measured Raman spectra and Raman images of the concretion at the points of 79 and 97 cm from the exposed sample surface. The detail of the sample is described in Muramiya et al.^4^.

### Methods

Raman images of 101-point square with 4 μm step were developed from the collected Raman spectra by use of the Raman imaging system (LabRAM Soleil, HORIBA). which consisted of 532-nm diode laser, a spectrometer, a 600-g/mm grating, a CCD camera (Synapse EMCCD Camera), and a microscope (Eclipse Ti2 Inverted Microscope, Nikon Co., Tokyo, Japan). The obtained Raman spectra of the concretion suffered from severe fluorescence, and thus we treated the spectra with SVD first, and then the smoothing was applied. Finally, baseline correction was carried out. To make baseline correction the fifth polynomial was used to approach a spectrum and then the approached spectrum was subtracted from the original spectrum The obtained Raman imaging data were decomposed by MCR and CLS methods (MVA Plus in LabSpec6, HORIBA).

Raman spectra in Fig. SI 3 were measured using a micro-Raman spectrometer (Raman-750, Seishin Syoji). The Raman spectra were collected with an excitation wavelength of 532 nm (50 mW) using a 100 ×/0.8NA objective lens (OLYMPUS) and 1/10 attenuation filter.

## Results and discussion

### Raman spectra and Raman imaging of the concretion

Figure SI 3a–c show the 532-nm excited micro-Raman spectra in the 700–100 cm^−1^ region, 1300–700 cm^−1^ region, and 1900–1300 cm^−1^ region of the concretion measured at a position located at 97 cm from the exposed surface, respectively. The Raman spectra of the concretion measured using the 532 nm excitation show very strong fluorescence, but its intensity depended on places. Bands at 1097, 299, and 176 cm^−1^ are easily assigned to ν_1_, ν_12_, and ν_13_ modes of dolomite, respectively. The frequencies and relative intensities of the bands are very close to those (1085, 281, 155 cm^−1^) of calcite, respectively^22,23^. This result is consistent with the fact that the concretion is composed mainly of dolomite. It also indicates that dolomite is a dominate component in the concretion. Thus, the present Raman results are in good agreement with the previous knowledge. The band assignment of a broad feature in the region of 1650–1550 cm^−1^ is uncertain. It can be seen from the result in Fig. SI 3 that one cannot obtain detailed information about the components in the concretion.

**Figure 1:**
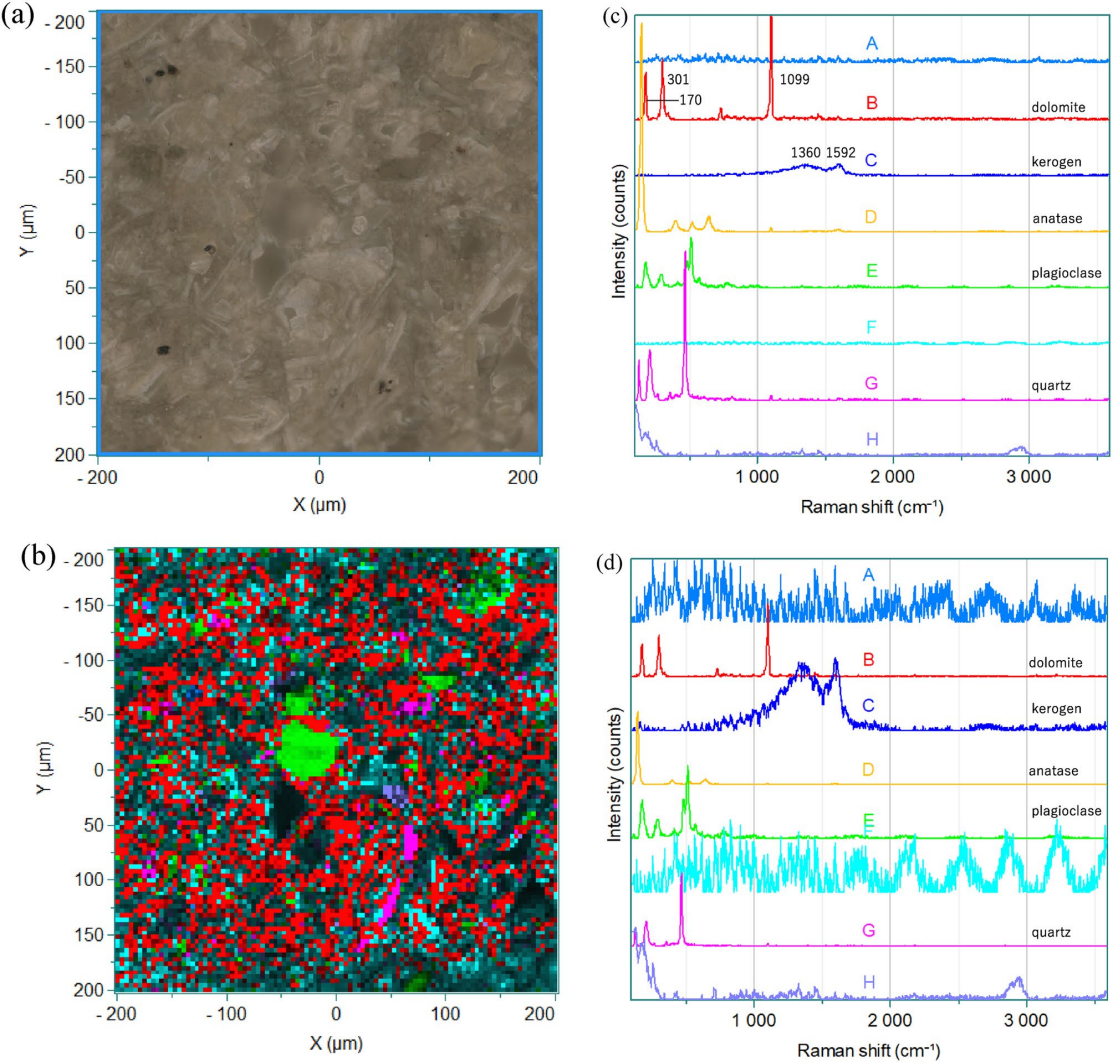
(**a**) An optical image and (**b**) by a Raman image of the concretion calculated by MCR. (**c**) Loading spectra obtained by MCR from the Raman image in (**b**). (**d**) Normalized loading spectra of those shown in (**c**).

Figure 1a,b display an optical image and a Raman image of the same place of the concretion. Comparison of Fig. 1a,b demonstrates the usefulness of visualization by Raman imaging. The Raman image can provide much clearer result about the distribution of various components of concretion. Figure 1c exhibits loading spectra obtained from Fig. 1b by MCR. Components were extracted from large amounts of mapping data by MCR and then, an image of the components was constructed. (A), (B), (C), (D), (E), (F), (G), (H) in Fig. 1c indicate the order of appearance of loading spectra. Figure 1d displays normalized loading spectra of those in Fig. 1c. The spectra were normalized by the most intense band in each spectrum.

It is noted that the MCR has nicely provided the loading spectra which are assigned to each component in the concretion. The colors of the spectra in Fig. 1c correspond to those in the Raman image shown Fig. 1b. Spectra in Fig. 1c (A) and (F) show strong fluorescence from components, and Raman bands could not be seen due to this strong fluorescence. Moreover, interference arising from the instrument affects the spectra especially above 2000 cm^−1^. A loading spectrum in Fig. 1c (B) shows four bands at 1099, 301 and 170 cm^−1^. These are characteristic of dolomite. The loading spectrum is very close to the spectra shown in Fig. SI 3. It can be seen from Fig. 1b that dolomite (red color) distributes dominantly from place to place. A loading spectrum in Fig. 1c (C) shows two intense bands at 1592 and 1360 cm^−1^. The spectra in Fig. 1c (C) and Fig. 1d (C) are very close to the reported spectra of kerogen, indicating the existence of kerogen in the concretion^24^. The image in Fig. 1b shows that kerogen (dark blue) distributes widely in the concretion.

The loading spectra in Fig. 1c (D), (E) and (G) are close to the reported spectra of anatase, plagioclase, and quartz, respectively. Judging from the Raman image it is very likely that the distributions of anatase, plagioclase, and quartz are rather limited. It is not easy to make assignments of bands in Fig. 1c (F), (H) and d (F), (H), but the distributions in Fig. 1b indicates these bands may be due to clastic sediments. The Raman image in Fig. 1b indicates that dolomite fills gaps of the sediments.

**Figure 2:**
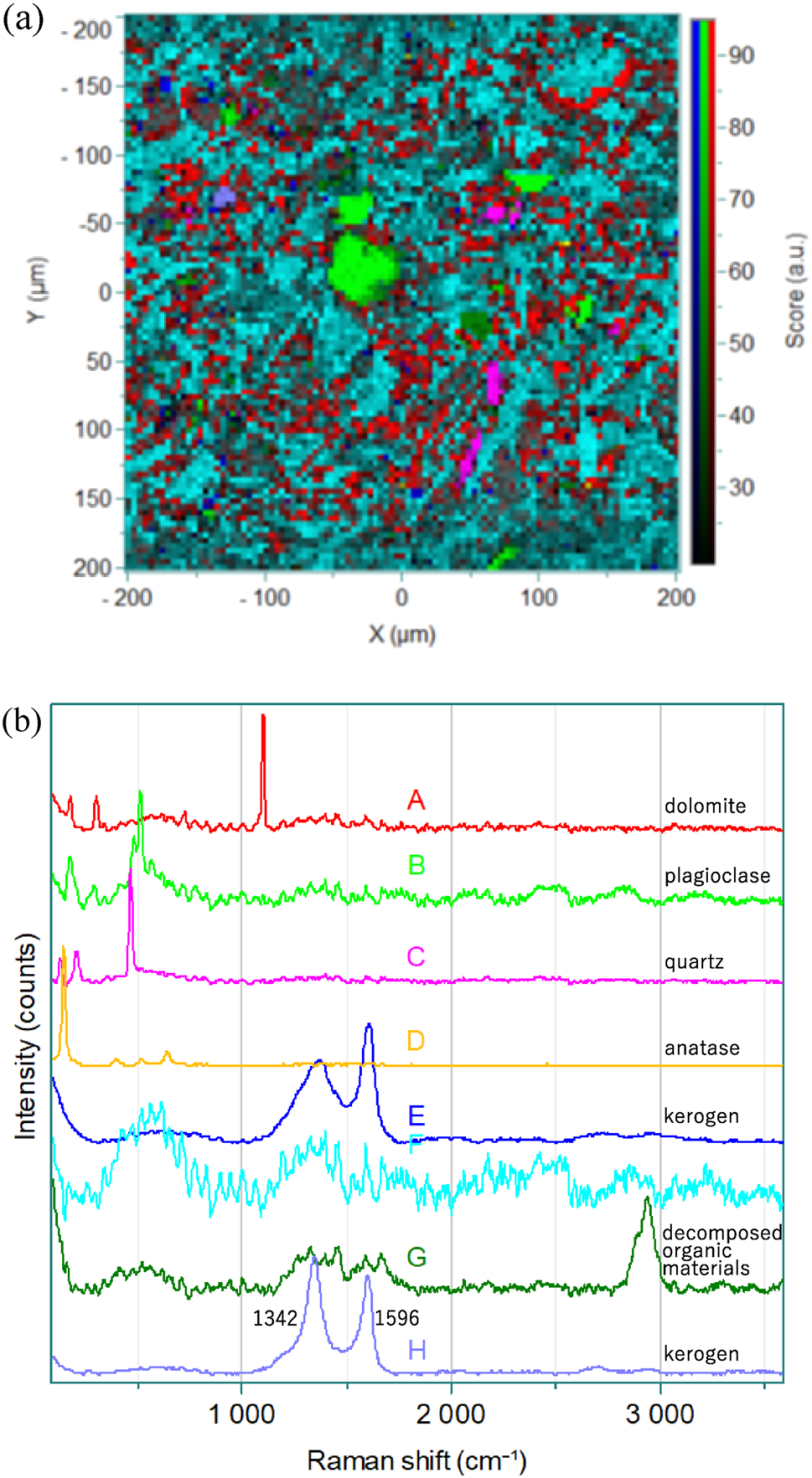
(**a**) A Raman image of the concretion calculated by CLS. (**b**) Loading spectra obtained by CLS from the Raman image in (**a**).

Figure 2a,b display another Raman image and the corresponding loading spectra, respectively, obtained from the same place as that for the image in Fig. 1b using CLS. In the case of CLS one can extract loading spectra voluntarily in any order. It is noted that the Raman images in Figs. 1b and 2b are very similar to each other but somewhat different. It is noted that MCR gives always the same results (the same image and the same loading plots) but in the case of CLS the results change with an operator. However, it may be possible for CLS to extract data which MCR cannot detect. A loading spectrum in Fig. 2b (A) is a spectrum that of dolomite. The wide distribution of this component is clearly recognized in the Raman image in Fig. 2b as in the case of that in the Raman image in Fig. 1b. Loading spectra in Fig. 2b (B), (C), and (D) are typical Raman spectra of plagioclase, quartz and anatase, suggesting that plagioclase, quarts and anatase are involved in the concretion. The Raman images indicates that the amounts of plagioclase, quartz and anatase are small. As in the case of Fig. 1c (C), a loading spectrum in Fig. 2b (E) is very close to a reported spectrum of kerogen.^24^ A band in Fig. 2b (F) is uncertain but it may be due to sediments. A spectrum in Fig. 2b (G) shows bands in the region of 3000–2800 cm^−1^ (CH stretching), and bands at ca. 1658 (amide I), ca. 1585, 1455(CH_2_ bending), 1323 (amide III), and 1261(amide III) cm^−1^. It is very likely that the spectrum arises from decomposed organic materials, which may come from corrosion by microorganisms. This may be an solid evidence for the existence of soft tissues with biological origin in the concretion. Various animals such as dinosaur and crayfish fossilize during decay and proteins remain^25^. Bands at 1596 and 1342 cm^−1^ in Fig. 2b (H) are due to G- and D-bands of carbon materials^24^, suggesting the existence of carbon materials in the concretion.

In this way the Raman images have provided very useful information about the visualization and distributions of components in the concretion which the optical image cannot yield. Comparison of MCR and CLS reveals that both methods are very useful but different advantages. MCR provides loading spectra in order but it does not give loading spectra of minor components such as decomposed organic materials and carbon materials while CLS shows loading spectra randomly but it may provide the spectra of minor components. Therefore, both methods are complementary each other. Raman imaging of geological materials, including concretions, can provide valuable information. Additionally, chemometrics techniques such as MCR and CLS can be combined with Raman imaging to derive insights that would be impossible to obtain with either method alone.

### Raman spectra measured from the points identified in Raman images

**Figure 3:**
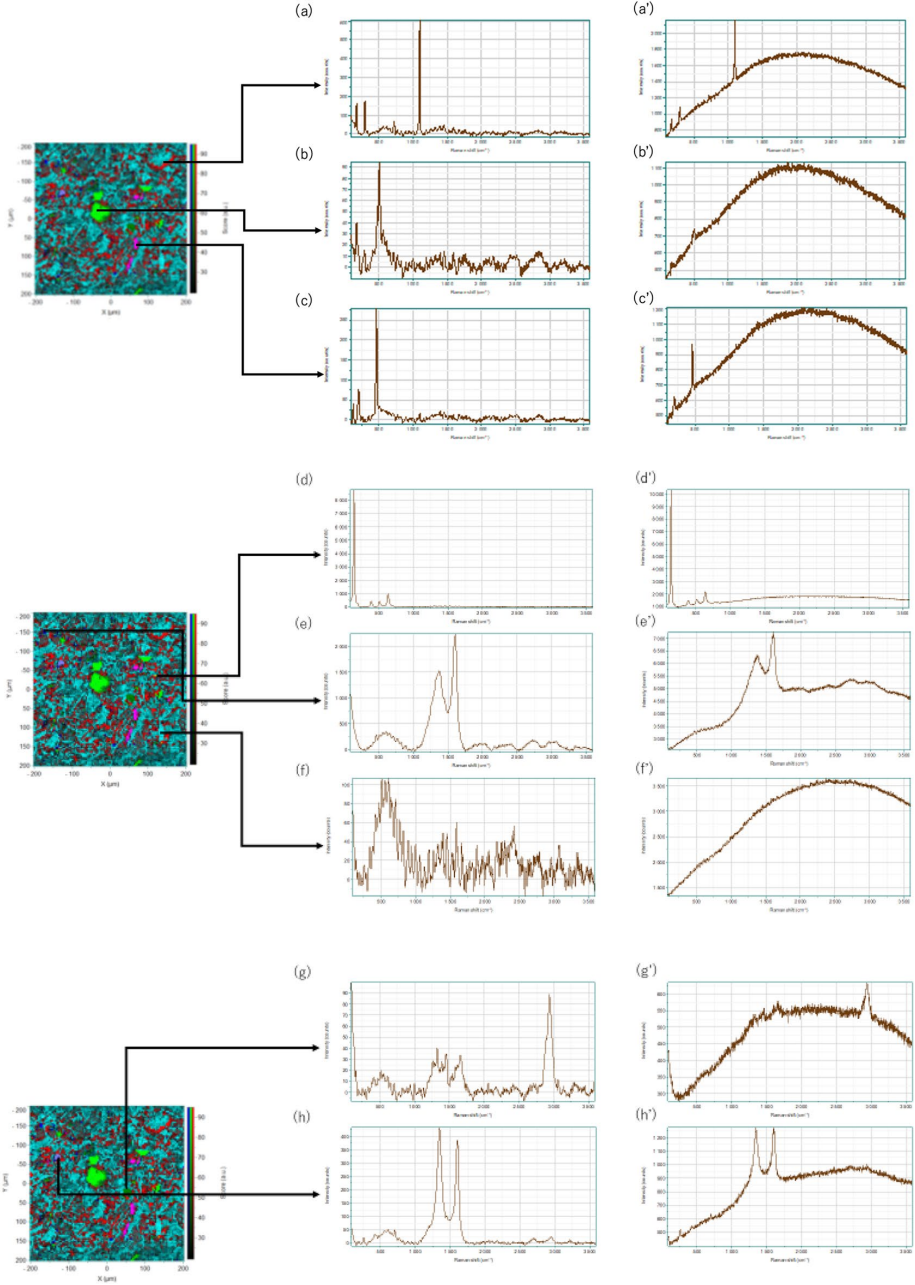
(**a**–**h**) 565-nm excited Raman spectra after pretreatments measured from the points in the Raman image in Fig. 2b. (**a**′–**h**′); the 565-nm excited Raman spectra without pretreatments measured from the same points in the Raman spectra in (**a**–**g**), respectively.

Figure 3a–g, and h exhibit 532-nm excited Raman spectra after pretreatments measured from the points in the Raman image in Fig. 2b. Figure 3a′–h′ depict the 532-nm excited Raman spectra without pretreatments measured from the same points as those in the Raman spectra in Fig. 3a–h, respectively. The spectra in Fig. 3a′–h′ show that the intensity of the fluorescence changes largely from point to point. The Raman spectra in Fig. 3a–g are very close to the spectra of dolomite, plagioclase, quartz, anatase, kerogen, collagen, and carbon materials, respectively. The results in Fig. 3 support those of the loadings spectra in Figs. 1b and 2b. The results in Figs. 1, 2, and 3 demonstrate that the combination of Raman spectra and Raman imaging is very useful to explore the identification and distribution of components in the concretion and is much more powerful than observation of an optical image with a polarizer.

### Clear evidences for the existence of soft tissues from biological origin in concretion and mechanism of the formation of concretion

**Figure 4:**
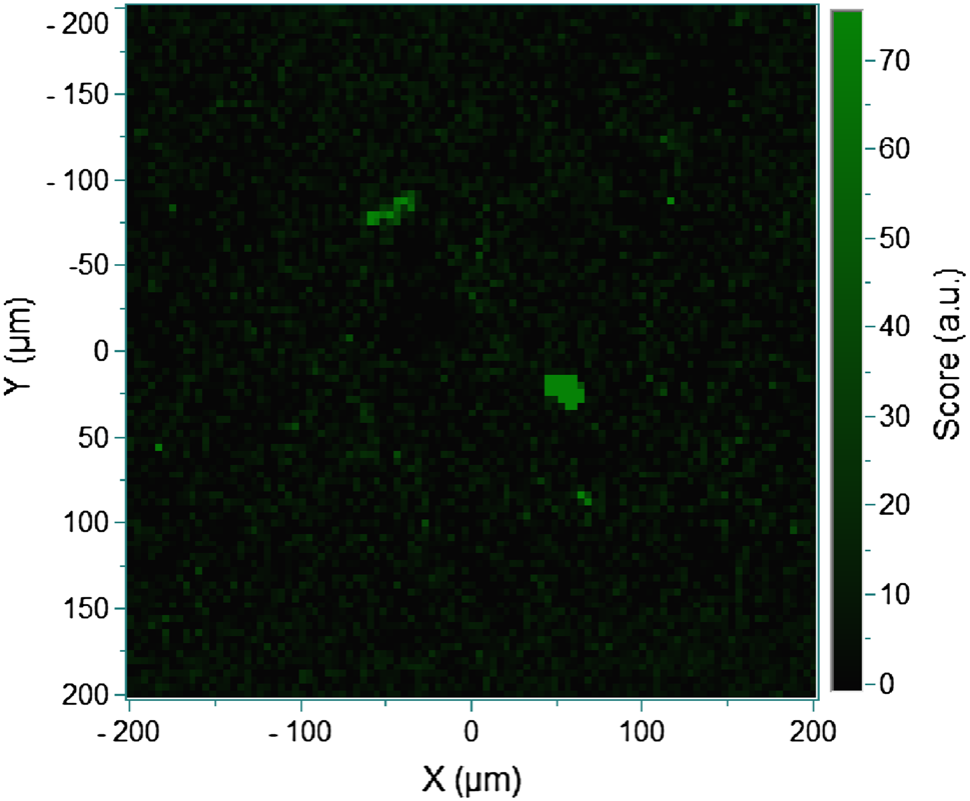
The intensity distribution of the 3000–2800 cm^−1^ region.

Figure 4 shows the intensity distribution of the 3000–2800 cm^−1^ region. As can be seen in Fig. 2b, only the loading spectrum (G) due to decomposed organic materials has an intense band in the 3000–2800 cm^−1^ region. Thus, Fig. 4 displays the distribution of decomposed organic materials. The green colored parts show rich distribution of decomposed organic materials. The present results clearly reveal the existence of carbon materials, kerogen, and one biological materials. Therefore, the present study suggests the concretion has biological origin. In contrast to previous indirect methods, such as elemental analysis or carbon and oxygen isotope analysis, the use of Raman imaging and chemometrics in this study provides direct evidence of biogenic origin, specifically through decomposed organic materials identification. The following steps might be considered for the process by which concretions are formed by the reaction of acids from organic matter of biogenic origin with calcium ions in seawater. (1) Decomposition of biological remains is progressed by biological microorganisms. (2) The rotten substance is not yet dissolved at the stage of (1), however, the tip of the substance is dissolved by changing into bicarbonate ions or polymers through the degradation process. Calcium carbonate is precipitated by supersaturation reaction with calcium ions. It is very difficult to clearly separate the processes (1) and (2), even in the case of reactions such as the rotting of present-day animals in seawater. A detailed Raman imaging in combination with MCR and CLS analysis will provide new evidence to understand this process in the future study.

## Conclusion

This study has demonstrated the potential of Raman imaging in combination with MCR and CLS in the investigation of concretion. The Raman images have enabled to explore the visualization of components in the concretion. Dolomite and kerogen appear in the image as major components; dolomite fills gaps of the clastic sediments. Of note is that even minor components such as plagioclase and decomposed organic materials are observed in the images. The calculations of loading spectra calculated by MCR and CLS have allowed us to identify the components included in the concretion. The results of Raman images and loading spectra obtained by MCR and CLS are similar to each other, but only the loading spectra obtained by CLS have provided the spectra of decomposed organic materials and carbon materials. This study presents direct evidence that dolomite concretions are of biogenic origin. The significant dimensions of the concretion imply that the organism it derives from possibly belonged to the category of gigantic marine animals. The combined technique of Raman imaging and chemometrics will be useful for investigating not only for concretions but also for fossils, rocks, and other geological samples. This approach can provide new insights that could be previously unobtainable.

### Supplementary Information

Supplementary Figures.

## Data Availability

The datasets analysed during the current study are available from the corresponding author M. Tsuboi (tsuboimot@kwansei.ac.jp) on reasonable request.
