# Supplementary material for: Visualization and identification of components in a gigantic spherical dolomite concretion by Raman imaging in combination with MCR or CLS methods
Source: Sci Rep. 2024 Jan 7;14:749. doi: 10.1038/s41598-024-51147-y (PMC10772084; doi:10.1038/s41598-024-51147-y)
Supplement: Supplementary file 1 — Supplementary Figures. [file 41598_2024_51147_MOESM1_ESM.pdf]

Figures in SI

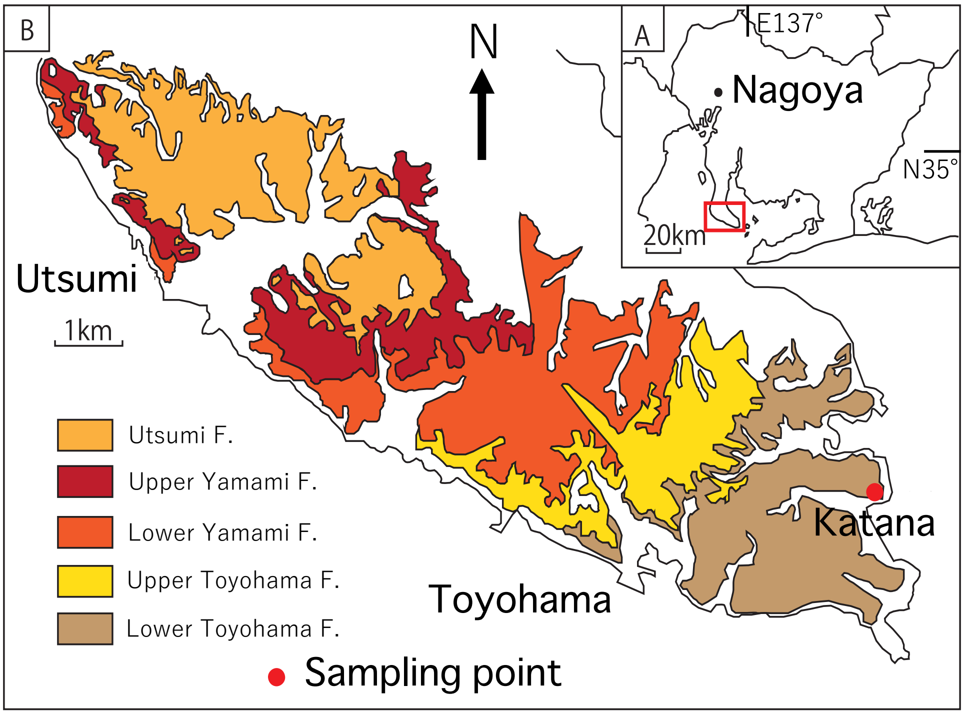

Figure SI 1 Geological map of the study area from Muramiya (2020)[4] and Kondo and Kimura (1987).[26] This map was prepared by tracing geological map based on Muramiya (2020)[4] and Kondo and Kimura (1987) [26] using commercial software. (A) The location of the Chita Peninsula, southwestern Japan. (B) Geological map of the Chita Peninsula, enlarged from the red square in Figure (A).

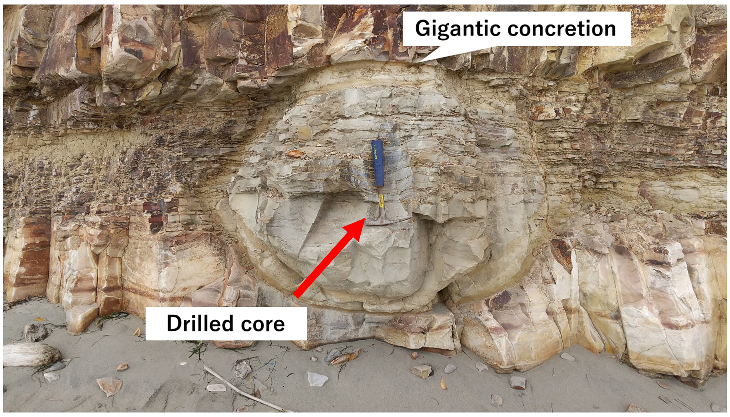

Figure SI 2　A photo of the concretion investigated. A red arrow indicates a drilled point.

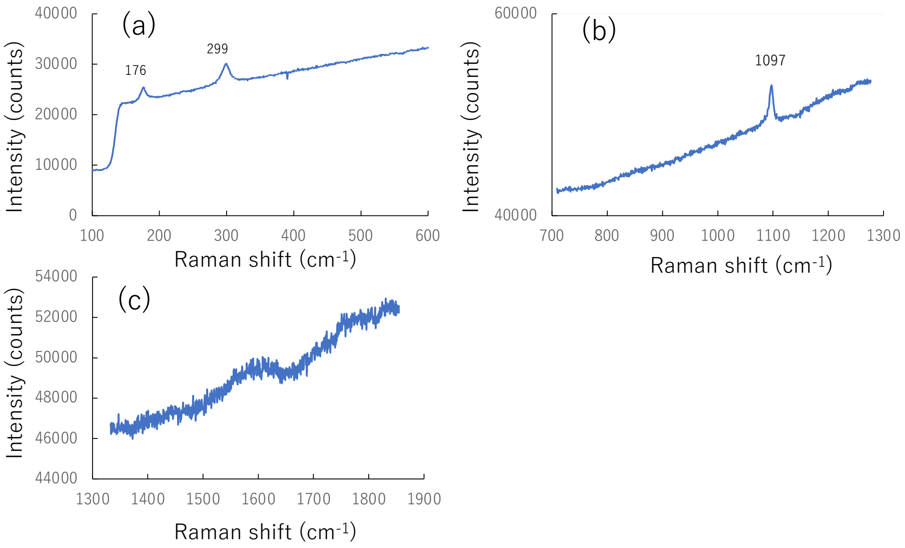

Figure SI 3 Raman spectra in the (a) 600-100, (b) 1300-700, and (c) 1900-1300 cm^{-1} regions of a point of the concretion.
